# Mental health literacy as a function of remoteness of residence: an Australian national study

**DOI:** 10.1186/1471-2458-9-92

**Published:** 2009-03-27

**Authors:** Kathleen M Griffiths, Helen Christensen, Anthony F Jorm

**Affiliations:** 1 Centre for Mental Health Research, The Australian National University, Canberra, ACT, 0200, Australia; 2 ORYGEN Research Centre, Department of Psychiatry, University of Melbourne, Locked Bag 10, Parkville, Victoria, 3052, Australia

## Abstract

**Background:**

Although there have been many population studies of mental health literacy, little is known about the mental health literacy of people who reside in rural areas. This study sought to determine the impact of remoteness on public knowledge of depression and schizophrenia.

**Methods:**

The mental health literacy of residents of major cities, inner regional, and outer-remote (including outer regional, remote, and very remote) regions were compared using data from a 2003–04 Australian national survey of the mental health literacy of 3998 adults. Measures included the perceived helpfulness of a range of professionals, non-professionals and interventions, and the causes, prognosis, and outcomes after treatment for four case vignettes describing depression, depression with suicidal ideation, early schizophrenia and chronic schizophrenia. Participant awareness of Australia's national depression initiative and depression in the media, their symptoms of depression and exposure to the conditions depicted in the vignettes were also compared.

**Results:**

Mental health literacy was similar across remoteness categories. However, inner regional residents showed superior identification of the disorders depicted in the suicidal ideation and chronic schizophrenia vignettes. They were also more likely to report having heard of Australia's national depression health promotion campaign. Conversely, they were less likely than major city residents to rate the evidence-based treatment of psychotherapy helpful for depression. Both inner regional and outer-remote residents were less likely to rate psychologists as helpful for depression alone. The rural groups were more likely to rate the non-evidence based interventions of drinking and painkillers as helpful for a depression vignette. In addition, outer-remote residents were more likely to identify the evidence based treatment of antipsychotics as harmful for early schizophrenia and less likely to endorse psychiatrists, psychologists, social workers and general practitioners as helpful for the condition.

**Conclusion:**

Mental health awareness campaigns in rural and remote regions may be most appropriately focused on communicating which interventions are effective for depression and schizophrenia and which mental health and other professionals are trained in the best-practice delivery and management of these. There is also a need to communicate to rural residents that alcohol and pain relievers are not an effective solution for depression.

## Background

Over the past decade it has been increasingly recognised that improving community *mental health literacy *may play an important role in increasing help seeking and addressing the high level of unmet need in the treatment of mental disorders [1-3]. Mental health literacy refers to 'knowledge and beliefs about mental disorders which aid their recognition, management or prevention' and includes the ability to 'recognise specific disorders; knowing how to seek mental health information; knowledge of risk factors and causes, of self-treatments, and of professional help available'[1].

There have been a large number of population studies of mental health literacy [4] and many countries have introduced initiatives designed to increase public knowledge about mental disorders [5,6]. However, there are very few published studies of the mental health literacy of rural residents. This is surprising given that the weight of evidence suggests that suicide rates are higher in rural than metropolitan areas in most regions of the world [7,8]. Moreover, it is important to identify any unique gaps in mental health literacy among rural residents in order to tailor the content of public awareness messages to the needs of this group. For example, Australia's national depression initiative *beyondblue *has recently initiated a campaign targeted at residents in rural areas asserting that rural residents are less aware of depression than urban residents [9]. Such assumptions require empirical substantiation and deconstruction if they are to guide rural health promotion programs.

To date there have been very few quantitative studies of the mental health literacy of rural residents [10-13] and, to our knowledge, only two published comparative studies of the mental health literacy of rural and city residents [12,13]. The first compared the depression literacy of residents in the State of South Australia according to their remoteness from service centres. The authors found no difference in levels of recognition of a depression vignette according to remoteness. Nor were there differences in the degree to which groups differing in remoteness considered antidepressants helpful or harmful for depression. However, the more remote residents in the study were more likely to consider psychologists, social workers, counsellors and telephone counselling harmful for depression. The other direct comparative study of mental health literacy among rural and city residents was conducted in the province of Alberta, Canada. The authors reported that rural residents were less likely than their geographically adjacent city counterparts to attribute schizophrenia to biological factors [13]. Such findings, if replicated, and generalisable to other rural regions, have implications for future mental health promotion initiatives in rural areas.

The aim of the current study was to compare the mental health literacy of rural and city residents in a national household survey of mental health literacy. We have previously documented the mental health literacy of the national sample in a series of publications on the recognition of mental disorders [14], perceptions about the helpfulness of treatments [14] and beliefs about the causes of different types of mental illness [15]. The current paper focuses specifically on the comparative results for the rural and city populations, and on the implications of the findings for designing promotion programs targeted at rural populations.

## Methods

Data were collected from a clustered national household survey of 3998 Australian adults aged 18 years or over during 2003–2004. The survey has been described in detail in previously published papers [14-16]. Sampling covered 250 census districts, all states and territories and both rural and metropolitan areas. Interviewers made up to five call backs to metropolitan areas and three call backs to rural areas. Of the 3998 respondents, 1001, 999, 997, and 1001 were presented with one of four vignettes depicting a DSM-IV mental disorder (see below). Response rate, computed as a percentage of the total number of contactable and physically available qualified respondents was 34%. The postcode for each respondent's place of residence was used to classify their locality according to the 2001 Australian Standard Geographical Classification (AGSC). The AGSC was developed by the Australian Bureau of Statistics as an indicator of the remoteness of a locality [17]. This system classifies localities by deriving an index based on their geographical distance to the nearest of each of five categories of centre which vary in population size. The resulting index is used to assign a locality into one of five remoteness categories: Major Cities, Inner Regional, Outer regional, Remote, and Very Remote. To ensure adequate sample sizes in the current study, the AGSC Outer regional, Remote and Very Remote categories were combined into a single remoteness category which will be designated here as outer-remote. All analyses of mental health literacy as a function of remoteness were based on these three categories.

### Survey interview

Respondents to the survey were each presented with a vignette which satisfied the DSM-IV and ICD criteria for either (i) a major depressive disorder; (ii) a major depressive disorder and suicidal thoughts; (iii) early schizophrenia; or (iv) chronic schizophrenia (see Appendix). The two vignettes involving major depressive disorder were identical except one involved a person with suicidal thoughts whereas the other did not. For each condition half of the vignettes employed a male character (John) and the remainder a female version (Mary). The resulting eight vignette types were randomly assigned to interviewees.

Respondents were asked a series of questions about the disorder depicted in the vignette. These included an open ended question to ascertain if they recognised the mental disorder in the vignette (What would you say, if anything, is wrong with John (Mary)?), their beliefs about whether each of a series of named sources of help and interventions would be 'helpful, harmful, or neither for John (Mary)' and the likelihood of improvement with and without professional help (full recovery with no further problems; full recovery, but problems would probably re-occur; partial recovery; partial recovery, but problems would probably re-occur; no improvement; get worse; don't know). They were also asked about long term specific outcomes for the person in the vignette if they received appropriate help (e.g., 'After getting help, how likely is he (she) to be violent?' – 'more likely, just as likely, less likely, depends') and about the causes of the disorder ('How likely do you think each of the following is to be a reason for such problems?' – 'very likely, likely, not likely, depends, don't know'). Respondents' exposure and personal experience of mental illness was ascertained by asking if they had 'ever had problems similar' to those of the character in the vignette, if a member of their 'family or close circle of friends' ever had such problems and if they had 'ever had a job that involved providing treatment or services' to a person with a problem like the character in the vignette ('yes, no, don't know'). Respondents were also questioned about their awareness of depictions of depression in the media ('Have you seen, read or heard anything in the media about depression in the last 12 months' – 'yes, no, don't know') and if they had heard of Australia's national depression initiative, *beyondblue *or a fictitious organisation called the "Mellow Yellow Institute" ('yes, no, don't know'). The survey also included a measure containing 12 items asking the participant to self-rate their current health status ('In the past month have you suffered from any of the following: colds, sore throats, headaches, dizziness, palpitations, breathlessness, backache, flu, anxiety, depression, tiredness, irritability, nervousness' – 'yes, no, don't know') [18]. The anxiety, depression, irritability and nervousness items comprise the 4NS, a brief measure of psychological distress which correlates 0.62 with the Present State Examination [18]. However, in the current study the 4NS was analysed on an item by item basis.

The socio-demographic characteristics collected in the survey included sex (male, female), educational background (dichotomised into Bachelors Degree, Not Bachelors degree), age (1 = 18–19 years, 2 = 20–24 years, 3 = 25–29 years, 4 = 30–34 years, 5 = 35–39 years, 6 = 40–44 years, 7 = 45–49 years, 8 = 50–54 years, 9 = 55–59 years, 10 = 60–64 years, 11 = 65–69 years, 12 = 70–74 years, 13 = 75+ years) and, as noted above, the postcode of the respondent's residence. The survey also incorporated social distance and stigma scales, but the results of these are published elsewhere [16,19].

Ethics approval for the study was granted by the Human Research Ethics Committee of The Australian National University.

### Statistical analysis

Percentages for each of the remoteness categories were calculated using the Complex Samples procedure in SPSS 16.0 which applies survey weights to provide more accurate population estimates. This procedure takes account of sampling weights and geographic clustering in the sample. Age was compared across remoteness categories using an Ordinal Logistic Regression for Complex Procedures and education and sex were compared using the Complex Samples Cross Tabs Procedure. Remoteness effects on mental health literacy were analysed using the Logistic Regression procedure for Complex Samples both controlling and not controlling for demographic variables (age, educational level and sex). Responses to items containing multiple response categories were dichotomised prior to analysis. (Recognition: 0 = incorrect, 1 = correct; Helpfulness of sources/intervention: 0 = harmful/don't know, 1 = helpful; Harmfulness of sources/interventions: 0 = don't know/helpful, 1 = harmful; Prognosis: 0 = no improvement/get worse/don't know, 1 = full recovery/partial recovery with no further problems, full/partial recovery but problems would probably re-occur; Long term specific outcomes: 0 = just as likely/less likely/depends, 1 = more likely; Causes: 0 = not likely/depends/don't know; 1 = likely/very likely; Experience with illness/awareness of initiatives/media/4NS items: 0 = no/don't know; 1 = yes).

The above procedures generated Odds Ratios (ORs) and 95% confidence intervals for the ORs. In each case, 'Major City' served as the standard reference such that an OR in excess of 1 indicated that the inner regional or outer-remote Australians were more likely to endorse an attribute. No adjustments were made for multiple comparisons. Accordingly, the emphasis in reporting the results is on the patterns of findings.

## Results

### Demographic status

Table 1 summarises the weighted age, sex and educational status for the major cities, inner regional and outer-remote groups. There was no significant difference in the distribution of males and females among the three groups (Likelihood ratio adjusted F(1.99, 494.39), p = 0.76). Nor was there a significant difference in the age of outer-remote and major city residents (Cumulative OR = 0.99, 95% CI 0.75–1.30). However, overall, the inner regional residents were marginally younger than the major city residents (Cumulative OR = 0.86; 95% CI = 0.66–0.99). In addition, the inner regional and outer-remote groups were less likely than the major cities group to have completed a Bachelor's degree (Likelihood ratio adjusted F(1.93, 478.25), p < .001).

**Table 1:** Demographic characteristics (weighted) for category of remoteness.

	Maj city*	**IR**	**ORe/R/VR**
Age			
1 = 18–19 years	3.9% (95% CI = 3.0–3–5)	4.1% (95% CI = 2.7–6.4)	3.1% (95% CI = 1.3–7.1)
2 = 20–24 years	9.1% (95% CI = 7.8–10.7)	8.2% (95% CI = 6.1–10.8)	8.3% (95% CI = 5.0–13.5)
3 = 25–29 years	8.1% (95% CI = 7.0–9.4)	5.9% (95% CI = 4.1–8.3)	9.2% (95% CI = 6.5–12.8)
4 = 30–34 years	11.6% (95% CI = 10.2–13.0)	9.1% (95% CI = 7.1–11.5)	12.9% (95% CI = 8.9–18.4)
5 = 35–40 years	9.5% (95% CI = 8.4–10.7)	9.6% (95% CI = 7.6–12.0)	8.6% (95% CI = 6.0–12.1)
6 = 41–44 years	10.4% (95% CI = 9.2–11.8)	10.2% (95% CI = 8.3–12.6)	12.0% (95% CI = 7.8–17.9)
7 = 45–50 years	9.6% (95% CI = 8.4–10.9)	10.1% (95% CI = 8.2–12.4)	7.4% (95% CI = 5.1–10.5)
8 = 51–54 years	8.5% (95% CI = 7.4–9.7)	8.4% (95% CI = 6.7–10.4)	8.6% (95% CI = 6.2–12.0)
9 = 55–59 years	7.3% (95% CI = 6.3–8.3)	7.1% (95% CI = 5.7–8.9)	6.6% (95% CI = 4.6–9.4)
10 = 60–64 years	5.8% (95% CI = 4.9–7.0)	7.4% (95% CI = 5.8–9.5	6.5% (95% CI = 4.2–9.7)
11 = 65–69 years	3.9% (95% CI = 3.2–4.7)	6.1% (95% CI = 4.9–7.5)	5.8% (95% CI = 4.0–8.3)
12 = 70–74 years	4.7% (95% CI = 4.0–5.6)	5.3% (95% CI = 4.0–7.1)	3.9% (95% CI = 2.6–5.9
13 = 70+ years	7.6% (95% CI = 6.3–9.1)	8.3% (95% CI = 6.3–11.0)	7.2% (95% CI = 4.2–11.8)
			
Sex (males)	49% (95% CI = 46.8–51.2)	48.1% (95% CI = 44.7–51.5)	50.6% (95% CI = 45.2–50.6)
Education	27.3% (95% CI = 24.5–30.4)	15.7% (95%CI = 12.3–19.8)	7.8% (95% CI = 4.8–12.7)

Maj City = Major City, IR = Inner Regional, ORe/R/VR = Outer Regional/Remote/Very remote; *Reference category; CI = Confidence interval.

### The effect of locality on mental health literacy

Mental health literacy for each locality is reported in Table 2, Table 3 and Table 4. Since the pattern of findings for the unadjusted and adjusted ORs was very similar, only the unadjusted ratios are reported. However, the statistical significance for both adjusted and unadjusted ORs is depicted in the tables (see footnote key).

**Table 2:** Percentage of respondents correctly labelling a vignette depicting mental disorders as a function of remoteness.

	**Depression**		**Depression with suicidal ideation**		**Early schizophrenia**		**Chronic schizophrenia**	
	%	Odds ratio (95% CI)	%	Odds ratio (95% CI)	%	Odds ratio (95% CI)	%	Odds ratio (95% CI)

Maj City*	67.1		75.9		42.0		32.3	
IR	61.2	0.78 (0.53–1.13)	83.5	**1.60 (1.02–2.51) **	39.6	0.91 (0.64–1.30)	46.7	**1.84 (1.32–2.58) **
Ore/R/VR	61.2	0.78 (0.40–1.52)	72.6	0.84 (0.44–1.62)	38.1	0.85 (0.49–1.48)	38.7	1.33 (0.67–2.62)

Maj City = Major City, IR = Inner Regional, ORe/R/VR = Outer Regional/Remote/Very remote; *Reference category; CI = Confidence interval.

Note: **Bolded data: **Odds Ratios significant (p < .05).

**Table 3:** Percentage of city and rural respondents indicating source would be (i) 'helpful' or (ii) 'harmful' for the person in the vignette.

**(i) HELPFUL**	**Depression**		**Depression with suicidal ideation**		**Early schizophrenia**		**Chronic schizophrenia**	
	%	Odds ratio (95% CI)	%	Odds ratio (95% CI)	%	Odds ratio (95% CI)	%	Odds ratio (95% CI)

**General practitioner**								

Maj City*	87.5		84.3		77.2		76.9	
IR	87.6	1.01 (0.61–1.67)	82.0	0.85 (0.56–1.29)	79.0	1.11 (0.76–1.62)	73.1	0.81 (0.55–1.20)
ORe/R/VR	85.1	0.82 (0.40–1.67)	88.5	1.43 (0.71–2.87)	65.3	**0.56 (0.34–0.91)**	79.8	1.19 (0.61–2.30)

**Pharmacist**								

Maj City*	34.4		31.3		23.7		29.2	
IR	38.8	1.21 (0.84–1.74)	34.2	1.14 (0.83–1.57)	22.9	0.95 (0.66–1.38)	23.9	0.76 (0.53–1.10)
ORe/R/VR	33.6	0.97 (0.62–1.52)	47.6	**2.00 (1.32–3.03)**	23.9	1.01 (0.64–1.59)	30.6	1.07 (0.68–1.70)

**Counsellor**								

Maj City*	83.8		86.0		84.9		84.3	
IR	81.4	0.84 (0.56–1.27)	84.5	0.89 (0.57–1.38)	86.7	1.17 (0.74–1.83)	79.0	0.70 (0.45–1.09)
ORe/R/VR	70.0	**0.45 (0.26–0.77)**	84.1	0.86 (0.50–1.49)	81.9	0.81 (0.44–1.46)	83.9	0.97 (0.57–1.65)

**Social worker**								

Maj City*	62.1		68.1		69.5		81.2	
IR	65.1	1.14 (0.82–1.58)	65.2	0.88 (0.62–1.23)	68.1	0.94 (0.65–1.36)	72.3	**0.61 (0.40–0.91)**
ORe/R/VR	61.8	0.99 (0.61–1.59)	65.5	0.89 (0.53–1.50)	59.1	**0.63 (0.44–0.92)**	79.7	0.91 (0.47–1.76)

**Phone counselling**								

Maj City*	64.4		65.1		56.1		47.0	
IR	63.9	0.98 (0.69–1.39)	67.1	1.09 (0.74–1.61)	58.7	1.11 (0.81–1.54)	46.1	0.96 (0.70–1.33)
ORe/R/VR	54.5	0.66 (0.42–1.04)	73.5	1.49 (0.82–2.71)	55.1	0.96 (0.60–1.55)	55.5	1.41 (0.80–2.49)

**Psychiatrist**								

Maj City*	67.1		72.9		81.6		82.2	
IR	60.4	0.75 (0.53–1.06)	67.5	0.78 (0.54–1.10)	82.4	1.06 (0.69–1.62)	76.6	0.71 (0.46–1.10)
ORe/R/VR	59.5	0.72 (0.46–1.12)	67.5	0.78 (0.45–1.34)	65.0	**0.42 (0.24–0.75)**	72.9	0.58 (0.34–1.01)

**Psychologist**								

Maj City*	70.5		70.9		75.9		78.1	
IR	61.6	***0.67 (0.47–0.96)***	65.9	0.80 (0.54–1.17)	72.1	0.82 (0.55–1.22)	65.4	**0.53 (0.37–0.76)**
ORe/R/VR	50.5	**0.43 (0.25 0.72)**	69.5	0.94 (0.56–1.58)	57.6	**0.43 (0.25–0.74)**	74.1	0.80 (0.45–1.440

**Close family**								

Maj City*	69.2		65.0		62.4		63.0	
IR	67.6	0.93 (0.62–1.39)	65.7	1.03 (0.71–1.51)	65.6	1.15 (0.84–1.57)	61.8	0.95 (0.65–1.40)
ORe/R/VR	56.7	0.58 (0.32–1.08)	60.5	0.83 (0.47–1.44)	57.2	0.81 (0.52–1.25)	46.8	***0.52 (0.28–0.97)***

**Close friends**								

Maj City*	77.3		77.5		71.8		72.5	
IR	81.5	1.29 (0.84–1.98)	76.0	0.92 (0.62–1.37)	77.9	1.39 (0.97–1.99)	71.2	0.94 (0.64–1.37)
ORe/R/VR	76.9	0.98 (0.50–1.92)	75.6	0.90 (0.52–1.54)	69.2	0.88 (0.53–1.47)	70.0	0.89 (0.51–1.54)

**Naturopath/herbalist**								

Maj City*	33.5		30.3		21.7		19.3	
IR	40.5	1.36 (0.93–1.97)	34.3	1.20 (0.79–1.82)	29.3	**1.49 (1.02–2.18)**	18.6	0.96 (0.64–1.43)
ORe/R/VR	31.7	0.92 (0.57–1.50)	38.3	1.43 (0.90–2.26)	25.8	1.25 (0.72–2.18)	21.7	1.16 (0.73–1.85)

**Clergy**								

Maj City*	47.2		51.2		35.8		44.0	
IR	42.9	0.84 (0.62–1.14)	51.6	1.02 (0.76–1.36)	42.4	1.32 (0.99–1.77)	39.3	0.82 (0.58–1.17)
ORe/R/VR	35.8	0.62 (0.36–1.09)	56.1	1.22 (0.78–1.90)	34.7	0.95 (0.54–1.69)	43.9	1.00 (0.57–1.76)

**Psychiatric ward**								

Maj City*	17.5		21.2		32.0		36.8	
IR	12.7	0.69 (0.43–1.09)	17.4	0.79 (0.50–1.23)	30.7	0.94 (0.67–1.33)	42.3	1.26 (0.88–1.79)
ORe/R/VR	17.0	0.97 (0.43–2.19)	19.5	0.90 (0.51–1.59)	35.2	1.16 (0.70–1.90)	33.7	0.87 (0.54–1.41)

**Health educator**								

Maj City*	87.1		85.5		86.1		84.1	
IR	85.5	0.88 (0.54–1.43)	85.7	1.01 (0.62–1.66)	86.3	1.02 (0.64–1.63)	84.4	1.03 (0.62–1.70)
ORe/R/VR	87.1	1.00 (0.53–1.90)	90.1	1.55 (0.69–3.46)	86.8	1.07 (0.52–2.19)	79.7	0.75 (0.35–1.58)

**On-line expert**								

Maj City*	54.8		50.2		58.0		46.1	
IR	51.5	0.88 (0.65–1.19)	45.4	0.83 (0.56–1.22)	50.3	0.73 (0.55–0.98)	39.7	0.77 (0.55–1.08)
ORe/R/VR	51.6	0.88 (0.55–1.41)	56.2	1.28 (0.82–1.99)	45.9	0.62 (0.38–1.01)	47.3	1.05 (0.56–1.99)

**Deal with it alone**								

Maj City*	13.2		10.3		12.5		12.5	
IR	13.4	1.02 (0.61–1.71)	7.1	0.67 (0.36–1.23)	6.9	**0.52 (0.27–0.99)**	8.5	0.65 (0.36–1.18)
ORe/R/VR	11.5	0.85 (0.39–1.89)	11.7	1.56 (0.59–2.25)	14.3	1.16 (0.55–2.46)	15.2	1.26 (0.65–2.43)

**(ii) HARMFUL**	**Depression**		**Depression with suicidal ideation**		**Early schizophrenia**		**Chronic schizophrenia**	

	%	Odds ratio (95% CI)	%	Odds ratio (95% CI)	%	Odds ratio (95% CI)	%	Odds ratio (95% CI)

**General practitioner**								

Maj City*	0.5		1.0		2.4		2.2	
IR	0.2	-	1.9	-	2.5	1.04 (0.37–2.91)	3.9	1.81 (0.68–4.80)
ORe/R/VR	1.7	-	0.0	-	3.9	1.68 (0.52–5.46)	3.6	1.67 (0.36–7.69)

**Pharmacist**								

Maj City*	8.9		6.9		8.5		8.7	
IR	7.9	0.88 (0.35–2.21)	12.4	1.90 (0.98–3.68)	10.6	1.27 (0.72–2.24)	6.3	0.71 (0.34–1.50)
ORe/R/VR	9.4	1.06 (0.47–2.35)	7.1	1.03 (0.40–2.64)	3.2	0.36 (0.11–1.12)	9.0	1.04 (0.39–2.76)

**Counsellor**								

Maj City*	2.2		2.3		3.1		2.2	
IR	4.3	1.95 (0.81–4.70)	2.6	1.10 (0.46–2.64)	2.8	0.91 (0.36–2.29)	2.7	1.22 (0.38–3.87)
ORe/R/VR	7.8	**3.68 (1.07–12.63)**	0.5	0.23 (0.03–1.69)	3.2	1.03 (0.30–3.55)	3.7	1.73 (0.48–6.32)

**Social worker**								

Maj City*	4.4		5.9		4.6		2.3	
IR	3.7	0.84 (0.39–1.82)	4.4	0.73 (0.34–1.57)	3.2	0.68 (0.31–1.51)	4.5	2.01 (0.84–4.84)
ORe/R/VR	7.6	1.78 (0.63–5.02)	4.5	0.75 (0.24–2.32)	5.9	1.30 (0.46–3.62)	5.2	2.31 (0.67–8.01)

**Phone counselling**								

Maj City*	4.8		6.7		7.6		11.8	
IR	7.4	1.59 (0.82–3.09)	6.1	0.90 (0.39–2.08)	8.5	1.12 (0.59–2.13)	11.4	0.96 (0.56–1.67)
ORe/R/VR	12.0	**2.70 (1.09–6.67)**	2.9	*0.42 (0.16–1.10)*	4.9	0.62 (0.21–1.85)	5.1	0.41 (0.16–1.03)

**Psychiatrist**								

Maj City*	6.0		7.8		5.2		3.8	
IR	9.8	1.69 (0.85–3.39)	9.7	1.27 (0.72–2.22)	4.2	0.80 (0.32–1.98)	6.1	1.63 (0.83–3.21)
ORe/R/VR	9.8	1.70 (0.80–3.61)	6.8	0.87 (0.27–2.77)	9.3	1.88 (0.91–3.89)	7.6	2.07 (0.75–5.70)

**Psychologist**								

Maj City*	3.5		5.0		3.4		3.1	
IR	9.4	**2.90 (1.32–6.36)**	6.4	1.31 (0.64–2.69)	2.1	0.61 (0.22–1.74)	4.5	1.45 (0.63–3.38)
ORe/R/VR	7.7	2.32 (0.95–5.63)	4.0	0.80 (0.16–4.07)	4.8	1.43 (0.51–4.05)	4.9	1.59 (0.60–4.21)

**Close family**								

Maj City*	4.4		3.6		4.7		5.3	
IR	4.2	0.96 (0.46–1.99)	5.4	1.53 (0.72–3.26)	8.3	1.82 (0.98–3.40)	5.8	1.09 (0.57–2.06)
ORe/R/VR	11.3	**2.81 (1.27–6.23)**	4.3	1.20 (0.46–3.13)	6.1	1.31 (0.54–3.17)	4.0	0.74 (0.20–2.76)

**Close friends**								

Maj City*	2.2		2.5		2.1		3.9	
IR	1.4	0.63 (0.20–1.97)	2.9	1.16 (0.44–3.05)	5.3	**2.65 (1.17–6.01)**	1.7	0.42 (0.14–1.27)
ORe/R/VR	2.7	1.26 (0.26–6.05)	3.0	1.21 (0.36–4.04)	4.3	2.14 (0.73–6.30)	1.9	0.46 (0.12–1.83)

**Naturopath/herbalist**								

Maj City*	10.3		13.3		16.6		13.7	
IR	12.1	1.20 (0.67–2.17)	15.6	1.21 (0.78–1.88)	11.2	0.63 (0.39–1.02)	19.4	1.52 (0.93–2.48)
ORe/R/VR	16.2	1.69 (0.84–3.38)	6.7	0.47 (0.21–1.02)	13.1	0.76 (0.37–1.56)	13.8	1.00 (0.56–1.81)

**Clergy**								

Maj City*	7.5		9.2		12.9		10.3	
IR	8.4	1.13 (0.60–2.12)	9.6	1.05 (0.64–1.73)	7.0	**0.51 (0.28–0.94)**	11.0	1.08 (0.60–1.94)
ORe/R/VR	11.7	1.63 (0.54–4.92)	8.6	0.93 (0.48–1.80)	13.3	1.04 (0.53–2.05)	8.1	0.77 (0.34–1.71)

**Psychiatric ward**								

Maj City*	51.6		50.1		37.6		32.7	
IR	56.2	1.20 (0.83–1.74)	47.4	0.90 (0.64–1.26)	41.7	1.19 (0.87–1.62)	33.0	1.01 (0.72–1.43)
ORe/R/VR	60.0	1.41 (0.82–2.43)	47.2	0.89 (0.48–1.65)	42.5	1.23 (0.83–1.81)	37.4	1.23 (0.74–2.04)

**Health educator**								

Maj City*	1.5		2.2		1.6		1.6	
IR	0.7	0.44 (0.06–3.48)	1.4	0.61 (0.16–2.25)	0.8	0.52 (0.11–2.50)	1.7	1.07 (0.32–3.55)
ORe/R/VR	1.8	1.17 (0.24–5.73)	1.6	0.73 (0.17–3.24)	7.1	1.25 (0.28–5.60)	1.4	0.83 (0.10–6.76)

**On-line expert**								

Maj City*	13.8		16.7		11.1		16.0	
IR	15.3	1.13 (0.72–1.75)	17.5	1.06 (0.66–1.69)	18.3	**1.79 (1.20–2.66)**	22.9	**1.55 (1.07–2.25)**
ORe/R/VR	15.5	1.15 (0.64–2.08)	10.5	0.58 (0.24–1.40)	25.3	**2.71 (1.72–4.26)**	12.4	0.75 (0.40–1.39)

**Deal with it alone**								

Maj City*	63.7		73.0		69.1		67.7	
IR	65.5	1.08 (0.77–1.52)	79.0	1.39 (0.93–2.07)	73.2	1.22 (0.86–1.73)	70.9	1.17 (0.82–1.67)
ORe/R/VR	62.8	0.96 (0.61–1.52)	78.3	1.34 (0.77–2.32)	73.6	1.25 (0.68–2.28)	59.9	0.71 (0.43–1.20)

Maj City = Major City, IR = Inner Regional, ORe/R/VR = Outer Regional/Remote/Very remote; *Reference category; CI = Confidence interval;

Note: **Bolded data: **Odds Ratios significant with and without adjustment for demographic status (p < .05). ***Bolded italicised***: Odds Ratios significant (unadjusted only). *Italicised:* Odds Ratios significant (adjusted only)

**Table 4:** Percentage of respondents indicating intervention would be (i) 'helpful' or (ii) 'harmful' as a function of remoteness and vignette.

**(i) HELPFUL**	**Depression**		**Depression – suicidal ideation**		**Early schizophrenia**		**Chronic schizophrenia**	
	%	Odds ratio (95% CI)	%	Odds ratio (95% CI)	%	Odds ratio (95% CI)	%	Odds ratio (95% CI)

**Vitamins/minerals**								

Maj City*	50.8		57.9		49.2		66.0	
IR	47.3	0.87 (0.62–1.23)	55.3	0.90 (0.63–1.29)	52.7	0.80 (0.57–1.14)	68.8	1.14 (0.76–1.69)
ORe/R/VR	47.2	0.87 (0.50–1.52)	45.3	0.60 (0.35–1.04)	52.8	1.09 (0.65–1.84)	68.4	1.12 (0.64–1.95)

**Pain relievers**								

Maj City*	12.8		10.8		7.2		10.6	
IR	20.8	**1.79 (1.13–2.84)**	14.5	1.40 (0.86–2.27)	9.3	1.31 (0.75–2.30)	8.2	0.75 (0.42–1.36)
ORe/R/VR	15.5	1.25 (0.59–2.66)	20.1	**2.08 (1.12–3.86)**	2.1	*0.28 (0.07–1.14)*	11.9	1.13 (0.52–2.50)

**Antidepressants**								

Maj City*	47.5		51.7		51.6		41.9	
IR	45.0	0.90 (0.64–1.27)	52.4	1.03 (0.73–1.45)	46.1	0.80 (0.59–1.10)	45.1	1.14 (0.79–1.63)
ORe/R/VR	44.7	0.89 (0.53–1.50)	58.8	1.33 (0.83–2.15)	45.5	0.78 (0.52–1.19)	42.0	1.00 (0.63–1.59)

**Antibiotics**								

Maj City*	8.9		7.6		4.1		6.5	
IR	15.3	**1.86 (1.11–3.13)**	8.0	1.06 (0.59–1.92)	4.0	0.99 (0.41–2.41)	6.9	1.08 (0.52–2.22)
ORe/R/VR	10.4	1.19 (0.60–2.39)	10.2	1.39 (0.67–2.89)	2.7	0.65 (0.19–2.18)	5.0	0.77 (0.33–1.80)

**Sleeping pills**								

Maj City*	21.9		21.0		18.0		10.4	
IR	26.9	1.31 (0.87–1.98)	23.0	1.12 (0.78–1.61)	20.1	1.15 (0.71–1.87)	15.8	1.63 (0.93–2.83)
ORe/R/VR	32.8	1.74 (0.96–3.15)	26.4	1.35 (0.79–2.30)	13.4	0.70 (0.38–1.31)	10.2	0.98 (0.48–1.99)

**Antipsychotics**								

Maj City*	11.0		16.1		33.0		36.6	
IR	10.9	0.98 (0.54–1.79)	15.4	0.95 (0.60–1.49)	33.1	1.00 (0.71–1.42)	41.7	1.24 (0.89–1.73)
ORe/R/VR	14.4	1.36 (0.61–3.07)	23.4	1.59 (0.87–2.91)	33.4	1.02 (0.56–1.84)	42.6	1.29 (0.72–2.29)

**Tranquillisers**								

Maj City*	12.7		12.6		17.2		13.7	
IR	16.8	1.39 (0.85–2.28)	16.5	1.38 (0.87–2.19)	19.3	1.15 (0.75–1.76)	19.6	***1.54 (1.02–2.33)***
ORe/R/VR	15.3	1.25 (0.71–2.19)	16.9	1.42 (0.75–2.68)	10.6	*0.57 (0.31–1.06)*	16.9	1.29 (0.73–2.29)

**Physical activity**								

Maj City*	92.2		93.0		87.3		80.1	
IR	92.4	1.02 (0.56–1.86)	89.2	0.62 (0.36–1.05)	86.7	0.95 (0.61–1.45)	80.0	0.99 (0.63–1.57)
ORe/R/VR	89.2	0.69 (0.33–1.47)	96.6	2.11 (0.76–5.85)	90.3	1.35 (0.49–3.75)	73.6	0.69 (0.41–1.18)

**Read about people with problem**								

Maj City*	79.3		79.3		79.7		74.4	
IR	80.4	1.07 (0.72–1.60)	81.2	1.13 (0.74–1.74)	79.2	0.97 (0.64–1.49)	76.7	1.13 (0.79–1.63)
ORe/R/VR	76.1	0.83 (0.46–1.52)	80.9	1.11 (0.66–1.87)	79.5	0.99 (0.56–1.73)	71.9	0.88 (0.53–1.47)

**Get out more**								

Maj City*	85.9		89.8		86.6		77.1	
IR	88.8	1.30 (0.79–2.12)	91.3	1.19 (0.65–2.16)	88.3	1.18 (0.70–1.97)	76.4	0.97 (0.62–1.51)
ORe/R/VR	91.9	1.87 (0.89–3.92)	91.6	1.24 (0.58–2.63)	88.4	1.19 (0.57–2.46)	72.3	0.78 (0.46–1.31)

**Learn relaxation**								

Maj City*	84.8		85.5		77.5		69.6	
IR	82.3	0.83 (0.54–1.28)	85.2	0.98 (0.63–1.51)	79.0	1.10 (0.73–1.66)	70.6	1.05 (0.73–1.50)
ORe/R/VR	76.9	0.60 (0.31–1.14)	84.3	0.92 (0.41–2.06)	68.0	0.62 (0.36–1.08)	56.4	**0.57 (0.35–0.91)**

**Cut out alcohol**								

Maj City*	58.6		59.9		66.5		52.2	
IR	49.2	**0.68 (0.49–0.96)**	56.9	0.88 (0.64–1.23)	65.7	0.96 (0.68–1.37)	57.6	1.24 (0.88–1.76)
ORe/R/VR	52.3	0.77 (0.50–1.21)	67.1	1.37 (0.91–2.05)	63.7	0.88 (0.53–1.46)	51.4	0.97 (0.58–1.60)

**Psychotherapy**								

Maj City*	47.2		52.1		59.7		62.5	
IR	36.4	**0.64 (0.46–0.90)**	46.0	0.78 (0.57–1.07)	60.0	1.01 (0.70–1.46)	63.8	1.06 (0.71–1.57)
ORe/R/VR	37.9	0.68 (0.41–1.15)	47.3	0.82 (0.46–1.46)	50.8	0.70 (0.44–1.09)	56.0	0.76 (0.45–1.29)

**Hypnosis**								

Maj City*	22.3		24.3		29.4		31.7	
IR	23.6	1.08 (0.73–1.59)	19.3	0.75 (0.50–1.12)	29.4	1.00 (0.70–1.43)	29.0	0.88 (0.60–1.30)
ORe/R/VR	19.9	0.87 (0.54–1.40)	33.4	1.57 (0.92–2.66)	35.7	1.33 (0.81–2.18)	28.6	0.86 (0.47–1.58)

**ECT**								

Maj City*	6.6		7.3		6.3		7.0	
IR	3.1	0.45 (0.21–1.00)	7.3	1.01 (0.47–2.16)	6.5	1.04 (0.52–2.05)	5.1	0.72 (0.32–1.60)
ORe/R/VR	7.8	1.19 (0.41–3.48)	5.7	0.76 (0.24–2.39)	6.5	1.03 (0.36–2.97)	5.6	0.79 (0.34–1.84)

**Occasional drink**								

Maj City*	40.9		38.7		29.9		26.5	
IR	52.0	**1.56 (1.12–2.19)**	50.6	**1.63 (1.15–2.29)**	38.0	***1.44 (1.01–2.05)***	29.6	1.17 (0.79–1.73)
ORe/R/VR	54.4	**1.73 (1.04–2.86)**	44.1	1.25 (0.76–2.06)	22.6	0.68 (0.36–1.31)	27.6	1.06 (0.49–2.27)

**Special diet**								

Maj City*	47.3		45.8		41.7		40.4	
IR	51.1	1.16 (0.82–1.63)	41.2	0.83 (0.59–1.16)	45.3	1.16 (0.83–1.62)	35.8	0.82 (0.58–1.16)
ORe/R/VR	48.5	1.05 (0.61–1.80)	56.2	**1.51 (1.02–2.26)**	36.4	0.80 (0.49–1.30)	39.9	0.98 (0.57–1.69)

**Website (information)**								

Maj City*	58.1		55.2		58.9		44.1	
IR	56.9	0.95 (0.69–1.30)	52.6	0.90 (0.61–1.33)	55.9	0.89 (0.65–1.21)	41.2	0.89 (0.62–1.26)
ORe/R/VR	58.5	1.01 (0.70–1.46)	61.3	1.29 (0.84–1.97)	49.1	0.67 (0.44–1.03)	52.0	1.37 (0.70–2.69)

**Book (information)**								

Maj City*	68.0		63.8		71.6		59.1	
IR	70.1	1.10 (0.80–1.52)	65.6	1.08 (0.73–1.60)	67.4	0.82 (0.58–1.16)	57.6	0.94 (0.66–1.35)
ORe/R/VR	75.9	1.48 (0.77–2.84)	69.8	1.31 (0.77–2.23)	69.6	0.91 (0.58–1.43)	64.4	1.25 (0.72–2.18)

**(ii)HARMFUL**	**Depression**		**Depression – suicidal ideation**		**Early schizophrenia**		**Chronic schizophrenia**	

	%	Odds ratio (95% CI)	%	Odds ratio (95% CI)	%	Odds ratio (95% CI)	%	Odds ratio (95% CI)

**Pain relievers**								

Maj City*	40.2		37.9		39.6		32.7	
IR	32.4	0.71 (0.50–1.02)	36.9	0.96 (0.68–1.36)	36.5	0.87 (0.63–1.21)	35.7	1.15 (0.78–1.68)
ORe/R/VR	29.8	0.63 (0.32–1.23)	33.5	0.83 (0.52–1.31)	39.7	1.00 (0.57–1.78)	46.3	**1.77 (1.16–2.71)**

**Antidepressants**								

Maj City*	26.5		25.0		20.0		29.3	
IR	27.4	1.05 (0.73–1.50)	20.4	0.77 (0.49–1.21)	30.0	**1.72 (1.19–2.48)**	29.9	1.03 (0.70–1.51)
ORe/R/VR	36.3	1.58 (0.96–2.60)	17.3	*0.63 (0.39–1.02)*	25.3	1.36 (0.85–2.18)	27.5	0.91 (0.58–1.44)

**Antibiotics**								

Maj City*	41.0		40.9		36.0		35.5	
IR	32.8	0.70 (0.48–1.02)	31.1	**0.65 (0.46–0.94)**	34.6	0.94 (0.67–1.33)	41.4	1.28 (0.88–1.87)
ORe/R/VR	29.9	0.62 (0.31–1.21)	29.7	0.61 (0.34–1.10)	37.9	1.08 (0.72–1.63)	36.5	1.04 (0.74–1.47)

**Sleeping pills**								

Maj City*	49.7		52.6		52.1		59.1	
IR	49.7	1.00 (0.71–1.41)	44.4	0.72 (0.51–1.01)	51.5	0.98 (0.67–1.42)	59.6	1.02 (0.73–1.43)
ORe/R/VR	48.0	0.93 (0.60–1.46)	46.8	0.79 (0.52–1.20)	67.0	**1.87 (1.18–2.97)**	54.1	0.82 (0.47–1.42)

**Antipsychotics**								

Maj City*	49.1		42.5		22.6		25.3	
IR	48.1	0.96 (0.65–1.41)	38.6	0.85 (0.58–1.25)	25.7	1.18 (0.82–1.71)	24.1	0.94 (0.67–1.32)
ORe/R/VR	41.1	0.72 (0.46–1.14)	26.5	**0.49 (0.27–0.87)**	37.5	**2.05 (1.22–3.45)**	18.7	0.68 (0.32–1.43)

**Tranquillisers**								

Maj City*	59.3		60.1		46.3		54.9	
IR	62.5	1.14 (0.79–1.65)	58.6	0.94 (0.68–1.30)	48.5	1.09 (0.76–1.57)	57.3	1.10 (0.78–1.56)
ORe/R/VR	63.7	1.20 (0.81–1.80)	64.1	1.19 (0.68–2.07)	55.4	1.44 (0.96–2.16)	58.2	1.14 (0.65–1.99)

**Cut out alcohol**								

Maj City*	4.1		4.3		3.0		2.6	
IR	5.9	1.47 (0.69–3.17)	9.2	**2.25 (1.08–4.68)**	3.9	1.32 (0.51–3.41)	3.8	1.49 (0.65–3.43)
ORe/R/VR	6.7	1.69 (0.47–6.01)	2.6	0.60 (0.15–2.35)	1.8	0.60 (0.14–2.51)	1.6	0.61 (0.08–4.58)

**Psychotherapy**								

Maj City*	8.6		10.3		4.7		7.0	
IR	13.6	1.66 (0.98–2.84)	11.9	1.17 (0.69–1.96)	6.4	1.39 (0.69–2.80)	6.2	0.88 (0.41–1.91)
ORe/R/VR	11.4	1.36 (0.52–3.58)	9.3	0.89 (0.32–2.48)	12.6	**2.91 (1.42–5.96)**	12.5	1.90 (0.88–4.12)

**Hypnosis**								

Maj City*	16.2		19.8		12.4		16.3	
IR	19.0	1.21 (0.77–1.91)	23.1	1.21 (0.76–1.94)	12.9	1.05 (0.64–1.71)	17.8	1.11 (0.71–1.74)
ORe/R/VR	19.0	1.22 (0.67–2.21)	18.1	0.90 (0.39–2.09)	16.7	1.42 (0.77–2.60)	16.2	0.99 (0.49–1.99)

**ECT**								

Maj City*	68.1		66.4		60.4		63.4	
IR	73.3	1.29 (0.85–1.97)	66.0	0.98 (0.66–1.48)	68.7	1.44 (0.98–2.11)	70.7	1.39 (0.93–2.08)
ORe/R/VR	69.7	1.08 (0.59–1.98)	61.4	0.80 (0.49–1.33)	74.9	**1.95 (1.22–3.13)**	67.4	1.19 (0.69–2.07)

**Occasional drink**								

Maj City*	16.6		21.6		30.9		24.0	
IR	13.7	0.80 (0.52–1.23)	13.9	**0.58 (0.36–0.94)**	27.0	0.82 (0.58–1.17)	27.5	1.20 (0.83–1.73)
ORe/R/VR	9.7	0.54 (0.27–1.10)	11.6	*0.48 (0.22–1.02)*	28.0	0.87 (0.54–1.40)	29.7	1.34 (0.66–2.69)

**Special diet**								

Maj City*	8.4		9.5		8.0		6.3	
IR	5.4	0.62 (0.32–1.21)	9.4	0.99 (0.57–1.75)	6.7	0.83 (0.46–1.51)	8.3	1.34 (0.69–2.62)
ORe/R/VR	7.8	0.93 (0.42–2.06)	6.2	0.63 (0.25–1.64)	8.7	1.11 (0.45–2.76)	10.2	1.70 (0.65–4.46)

**Website (information)**								

Maj City*	14.6		16.1		12.2		18.3	
IR	14.3	0.97 (0.62–1.51)	14.4	0.88 (0.55–1.40)	13.9	1.15 (0.78–1.72)	24.5	***1.44 (1.01–2.08)***
ORe/R/VR	17.5	1.23 (0.73–2.09)	10.5	0.62 (0.27–1.41)	14.1	1.18 (0.73–1.90)	12.7	0.65 (0.35–1.22)

**Book (information)**								

Maj City*	7.8		9.6		6.3		8.9	
IR	6.6	0.84 (0.48–1.49)	7.8	0.79 (0.43–1.45)	9.4	1.55 (0.83–2.87)	11.4	1.33 (0.77–2.28)
ORe/R/VR	9.8	1.29 (0.63–2.63)	6.7	0.67 (0.36–1.27)	7.1	1.14 (0.55–2.37)	8.2	0.91 (0.32–2.60)

Maj City = Major City, IR = Inner Regional, ORe/R/VR = Outer Regional/Remote/Very remote; *Reference category; CI = Confidence interval; Note: **Bolded data: **Odds Ratios significant with and without adjustment for demographic status (p < .05). ***Bolded italicised***: Odds Ratios significant (unadjusted only). *Italicised:* Odds Ratios significant (adjusted only). Data omitted for variables with <6% endorsement.

#### Recognition

Table 2 shows the percentage of major city, inner regional and outer-remote residents who correctly identified the nature of the problem experienced by the person in the vignette. Residents living in inner regional Australia were more likely than residents in major cities to correctly identify the depression with suicidal ideation and chronic schizophrenia vignettes. The effect remained significant after adjusting for demographic status.

#### Perceived helpfulness and harmfulness of professionals, non-professionals and interventions

Tables 3 and 4 depict the percentage of major city, inner regional and outer-remote residents who indicated that a particular person or organisation or intervention would be 'helpful' or 'harmful' for the person in the vignette. There were few differences in the ratings made by residents from major cities compared to those made by more remote residents. Key differences which did emerge are reported below.

##### Sources of help

###### Depression vignettes

Compared to city residents, outer-remote residents were less likely to rate psychologists and counsellors as helpful for depression alone and more likely to rate close family as harmful. The inner regional group were also less likely to rate psychologists as helpful. Moreover, they more often believed psychologists were harmful.

###### Schizophrenia vignettes

Those living in outer-remote areas were less likely than city residents to rate a psychiatrist, psychologist, social worker or GP as helpful for early schizophrenia. The inner regional group were more likely than city residents to consider that close friends would be harmful for early schizophrenia but were less likely to endorse dealing alone with early schizophrenia. Inner regional residents were less likely than city residents to rate psychologists and social workers as helpful for chronic schizophrenia. Outer-remote residents were less likely to consider family as helpful for chronic schizophrenia.

##### Interventions

###### Depression vignettes

Both outer-remote and inner regional residents were more likely than major city residents to endorse taking an occasional drink for depression alone. In addition, inner regional residents were less convinced of the helpfulness of reducing alcohol intake for depression, more often endorsed pain relievers and antibiotics, and less often endorsed psychotherapy and ECT for depression. With respect to the depression with suicidal ideation vignette, inner regional residents were more likely to report that taking an occasional drink would be helpful, less likely to report that it would be harmful, and more likely to believe that restricting alcohol intake would be harmful. Outer-remote residents were more likely than city residents to rate pain relievers as helpful for depression and suicidal ideation and less likely to rate antipsychotics as harmful.

###### Schizophrenia vignettes

Outer-remote residents were more likely to believe that antipsychotic medications, psychotherapy, sleeping pills and ECT would be harmful for early schizophrenia. Inner regional residents were more likely to believe the occasional drink would be helpful and antidepressants harmful for early schizophrenia. With respect to chronic schizophrenia, the outer-remote residents were less likely to endorse relaxation training, and more likely to believe that pain killers would be harmful. The inner regional group more often endorsed tranquillisers.

#### Prognosis and outcomes

Table 5 (i) shows the percentage of major city, inner regional and outer regional/remote residents predicting improvement (full, partial, with or without reoccurrence) for the person in the vignette with and without professional help. There were very few differences between the groups. Inner regional residents were somewhat less likely than major city residents to believe that the person in the depression vignette with suicidal ideation would improve without professional input. However, a reverse trend was noted for depression alone.

**Table 5:** Percentage of respondents predicting (i) improvement ('full' or 'partial' recovery); and (ii) outcomes if treated as a function of remoteness.

	**Depression**		**Depression with suicidal ideation**		**Early schizophrenia**		**Chronic schizophrenia**	
	%	Odds ratio (95% CI)	%	Odds ratio (95% CI)	%	Odds ratio (95% CI)	%	Rural Odds ratio (95% CI)

**(i) Improvement**								

**With professional help**								

Maj City*	96.6		97.1		97.1		95.9	
IR	96.9	1.08 (0.48–2.43)	94.9	0.55 (0.25–1.22)	96.2	0.74 (0.29–1.91)	94.3	0.70 (0.33–1.50)
ORe/R/VR	95.2	0.69 (0.24–2.00)	93.9	0.46 (0.17–1.21)	95.2	0.58 (0.20–1.68)	92.1	0.49 (0.21–1.18)

**Without professional help**								

Maj City*	14.0		12.3		5.0		3.5	
IR	18.8	*1.43 (0.91–2.24)*	6.4	**0.49 (0.29–0.85)**	7.7	1.59 (0.82–3.09)	1.2	0.33 (0.09–1.17)
ORe/R/VR	20.1	1.55 (0.83–2.88)	13.7	1.13 (0.52–2.44)	9.7	2.05 (0.94–4.47)	3.0	0.84 (0.12–5.96)

**(ii) Outcomes if treated, compared to others in the community:**								

**Violent**								

Maj City*	5.8		6.5		12.3		9.0	
IR	4.8	0.83 (0.41–1.69)	1.9	**0.28 (0.10–0.76)**	13.0	1.07 (0.64–1.77)	11.8	1.35 (0.76–2.40)
ORe/R/VR	3.9	0.66 (0.20–2.22)	2.1	0.31 (0.08–1.19)	10.5	0.83 (0.40–1.75)	8.7	0.96 (0.38–2.41)

**Too much alcohol**								

Maj City*	13.0		14.5		15.1		10.2	
IR	17.7	1.44 (0.91–2.29)	12.8	0.87 (0.52–1.45)	13.8	0.90 (0.59–1.39)	13.9	1.43 (0.83–2.45)
ORe/R/VR	18.0	1.47 (0.77–2.82)	21.7	1.64 (0.83–3.26)	10.8	0.68 (0.31–1.51)	10.8	1.06 (0.49–2.32)

**Take illegal drugs**								

Maj City*	9.9		14.3		16.6		12.3	
IR	14.0	1.49 (0.91–2.43)	13.8	0.96 (0.60–1.54)	15.8	0.94 (0.63–1.40)	13.8	1.14 (0.70–1.87)
ORe/R/VR	17.7	**1.97 (1.01–3.85)**	21.4	1.63 (0.90–2.97)	9.7	0.54 (0.27–1.07)	7.4	0.57 (0.24–1.35)

**Poor friendships**								

Maj City*	13.6		15.2		24.9		25.9	
IR	8.7	*0.61 (0.37–1.00)*	16.7	1.12 (0.70–1.79)	25.7	1.04 (0.71–1.53)	25.2	0.96 (0.65–1.43)
ORe/R/VR	15.0	1.13 (0.54–2.35)	19.9	1.39 (0.68–2.88)	17.2	0.63 (0.38–1.04)	12.7	**0.42 (0.21–0.85)**

**More likely suicide**								

Maj City*	17.5		22.4		31.1		26.9	
IR	20.5	1.21 (0.83–1.77)	23.1	1.04 (0.71–1.52)	33.9	1.14 (0.82–1.57)	30.7	1.21 (0.81–1.79)
ORe/R/VR	20.7	1.23 (0.62–2.42)	28.1	1.35 (0.84–2.18)	33.7	1.13 (0.69–1.85)	26.2	0.97 (0.52–1.81)

**Understanding others' feelings (less likely)**								

Maj City*	57.3		61.4		47.2		40.1	
IR	60.1	1.12 (0.79–1.60)	64.6	1.15 (0.84–1.58)	48.3	1.05 (0.78–1.42)	32.3	**0.71 (0.53–0.95)**
ORe/R/VR	71.0	**1.83 (1.11–3.01)**	63.1	1.08 (0.62–1.87)	48.7	1.07 (0.66–1.73)	38.7	0.94 (0.60–1.47)

**Caring parent (less likely)**								

Maj City*	31.1		31.4		21.9		18.1	
IR	31.1	1.00 (0.70–1.44)	25.3	0.74 (0.50–1.09)	19.4	0.86 (0.59–1.25)	13.7	0.72 (0.42–1.22)
ORe/R/VR	36.0	1.25 (0.73–2.14)	28.4	0.87 (0.48–1.58)	16.1	0.69 (0.36–1.31)	19.6	1.10 (0.61–1.99)

**Productive worker (less likely)**								

Maj City*	26.3		24.8		17.3		16.9	
IR	28.2	1.10 (0.72–1.67)	21.1	0.81 (0.53–1.24)	13.2	0.72 (0.45–1.17)	13.9	0.80 (0.46–1.37)
ORe/R/VR	31.1	1.26 (0.77–2.07)	31.5	1.40 (0.84–2.34)	12.3	0.67 (0.29–1.53)	19.1	1.16 (0.60–2.24)

**Creative/artistic**								

Maj City*	21.2		22.9		27.9		24.5	
IR	19.9	0.93 (0.59–1.44)	17.9	*0.73 (0.51–1.05)*	23.	0.79 (0.57–1.08)	32.7	1.50 (1.00–2.25)
ORe/R/VR	21.3	1.01 (0.56–1.80)	25.2	1.13 (0.62–2.08)	19.3	0.62 (0.30–1.29)	31.2	1.40 (0.77–2.52)

Maj City = Major City, IR = Inner Regional, ORe/R/VR = Outer Regional/Remote/Very remote; *Reference category; CI = Confidence interval.

**Bolded data: **Odds Ratios significant with and without adjustment for demographic status (p < .05). ***Bolded italicised***: Odds Ratios significant (unadjusted only). *Italicised:* Odds Ratios significant (adjusted only).

Table 5 (ii) depicts the percentage of each group of residents predicting that various outcomes would be 'more likely' for the person in the vignette compared to the general community. There were few differences in beliefs about outcome as a function of remoteness and the status of these individual findings in the context of multiple significance tests is unclear.

#### Beliefs about cause

As shown in Table 6, there were few differences between the groups with respect to beliefs about the causes of the problems for the people depicted in the vignettes. It is of interest however that outer-remote residents were significantly more likely than city residents to attribute schizophrenia to genetic causes. Similarly, inner regional more often applied a genetic attribution to chronic schizophrenia.

**Table 6:** Percentage of city and rural respondents nominating different causes of each disorder as 'likely' or 'very likely'.

	**Depression**		**Depression with suicidal ideation**		**Early schizophrenia**		**Chronic schizophrenia**	
	%	Odds ratio (95% CI)	%	Odds ratio (95% CI)	%	Odds ratio (95% CI)	%	Odds ratio (95% CI)

**Virus or infection**								

Maj City*	48.4		39.4		31.7		33.4	
IR	52.5	1.18 (0.86–1.61)	42.5	1.14 (0.81–1.60)	35.0	1.16 (0.81–1.66)	33.5	1.01 (0.72–1.41)
ORe/R/VR	60.3	1.62 (0.95–2.77)	56.5	***2.00 (1.41–2.84)***	34.9	1.15 (0.68–1.96)	32.2	0.95 (0.63–1.43)

**Allergy**								

Maj City*	43.1		36.3		30.5		27.0	
IR	49.8	1.31 (0.91–1.87)	38.8	1.11 (0.78–1.59)	35.1	1.23 (0.87–1.75)	30.9	1.21 (0.85–1.72)
ORe/R/VR	53.4	1.51 (0.86–2.67)	41.8	1.26 (0.80–2.00)	35.1	1.23 (0.76–1.99)	31.7	1.26 (0.68–2.33)

**Day-to-day problems**								

Maj City*	96.6		95.9		88.7		86.7	
IR	98.2	1.96 (0.63–6.06)	95.3	0.87 (0.42–1.80)	92.3	1.54 (0.90–2.64)	85.3	0.89 (0.52–1.53)
ORe/R/VR	96.8	1.06 (0.24–1.65)	95.5	0.90 (0.37–2.18)	86.6	0.83 (0.40–1.74)	89.6	1.33 (0.62–2.86)

**Recent death close friend/relative**								

Maj City*	95.2		94.4		86.6		83.6	
IR	97.1	-	96.9	1.89 (0.85–4.23)	89.8	1.35 (0.86–2.13)	80.4	0.81 (0.51–1.29)
ORe/R/VR	100.0	-	95.6	1.29 (0.40–4.18)	84.1	0.82 (0.43–1.55)	84.2	1.05 (0.47–2.34)

**Recent trauma**								

Maj City*	93.2		92.2		85.8		82.4	
IR	96.8	2.24 (0.94–5.31)	95.8	1.92 (0.99–3.70)	90.0	1.50 (0.93–2.41)	80.7	0.89 (0.56–1.42)
ORe/R/VR	92.7	0.93 (0.34–2.57)	93.8	1.29 (0.53–3.14)	82.7	0.79 (0.40–1.56)	87.5	1.50 (0.70–3.19)

**Childhood problems**								

Maj City*	91.3		94.4		89.7		91.8	
IR	93.8	1.44 (0.77–2.69)	96.4	1.57 (0.67–3.65)	94.0	1.79 (0.96–3.33)	88.3	0.67 (0.37–1.22)
ORe/R/VR	89.4	0.80 (0.36–1.77)	99.0	6.02 (0.81–44.72)	87.9	0.84 (0.39–1.81)	96.6	2.56 (0.77–8.49)

**Genetic**								

Maj City*	67.9		69.9		71.4		74.9	
IR	66.4	0.94 (0.62–1.41)	65.0	0.80 (0.56–1.15)	68.0	0.85 (0.60–1.21)	67.1	***0.68 (0.48–0.97)***
ORe/R/VR	65.1	0.88 (0.57–1.37)	66.0	0.84 (0.47–1.50)	56.4	***0.52 (0.34–0.79)***	75.7	1.04 (0.52–2.09)

**Nervousness**								

Maj City*	66.5		63.5		57.3		56.6	
IR	69.4	1.14 (0.80–1.63)	66.9	1.16 (0.83–1.64)	60.4	1.13 (0.79–1.63)	56.6	1.00 (0.73–1.36)
ORe/R/VR	77.1	***1.70 (1.04–2.79)***	70.2	1.36 (0.77–2.40)	67.1	1.52 (0.83–2.78)	66.4	1.51 (0.85–2.69)

**Weakness of character**								

Maj City*	41.3		46.0		39.9		36.4	
IR	49.7	1.41 (1.00–1.98)	43.1	0.89 (0.65–1.22)	36.4	0.86 (0.62–1.20)	31.4	0.80 (0.56–1.14)
ORe/R/VR	47.6	1.29 (0.80–2.08)	43.2	0.90 (0.53–1.50)	46.4	1.30 (0.83–2.04)	35.6	0.97 (0.52–1.78)

Maj City = Major City, IR = Inner Regional, ORe/R/VR = Outer Regional/Remote/Very remote; *Reference category; CI = Confidence interval.

Note: **Bolded data: **Odds Ratios significant with and without adjustment for demographic status (p < .05). ***Bolded italicised:*** Odds Ratios significant (unadjusted only). *Italicised*: Odds Ratios significant (adjusted only).

### Effect of locality on awareness about depression initiatives

Table 7(i) shows the percentages of people in each remoteness category indicating that they had heard of *beyondblue *(Australia's national depression initiative), had heard of a fictitious organisation, 'The Mellow Yellow Institute', and had heard or read an item about depression in the media. Inner regional residents were somewhat more likely than major city residents to report having heard of *beyondblue*, although they were no more likely to report having heard of the fictitious institute.

**Table 7:** Percentage of participants reporting (i) awareness of initiatives (ii) psychological distress and (iii) experience of the disorder depicted in the vignette.

	**(i) Awareness**				**(ii) Psychological distress**			
								

	%	Odds Ratio (95% CI)			%	Odds Ratio (95% CI)		

	**Beyondblue**				**Anxiety**			

Maj City*	23.9				22.2			
IR	26.6	***1.37 (1.08–1.73)***			21.0	1.13 (0.95–1.36)		
ORe/R/VR	22.6	0.97 (0.58–1.63)			14.1	1.09 (0.86–1.38)		

	**Mellow Yellow Institute**				**Depression**			

Maj City*	8.9				15.3			
IR	10.8	1.15 (0.84–1.58)			15.8	1.20 (0.94–1.53)		
ORe/R/VR	5.0	0.89 (0.61–1.29)			13.4	1.23 (0.84–1.79)		

	**Media reports**				**Irritability**			

Maj City*	59.5				39.4			
IR	60.9	1.08 (0.87–1.33)			43.1	**1.25 (1.01–1.54)**		
ORe/R/VR	60.4	1.18 (0.97–1.43)			44.9	1.19 (0.91–1.57)		

					**Nervousness**			

Maj City*					19.0			
IR					18.7	1.06 (0.85–1.33)		
ORe/R/VR					15.2	1.02 (0.76–1.37)		

								

	**(iii) Experience with disorder depicted in vignette**							

	**Depression**		**Depression + suicidal ideation**		**Early schizophrenia**		**Chronic schizophrenia**	

	%	Odds Ratio (95% CI)	%	Odds Ratio (95% CI)	%	Odds Ratio (95% CI)	%	Odds Ratio (95% CI)

	**Family/friend with problem**							

Maj City*	60.9		59.8		48.2		35.4	
IR	64.8	1.18 (0.83–1.68)	66.3	*1.32 (0.96–1.83)*	52.4	1.18 (0.89–1.57)	44.6	***1.47 (1.04–2.07)***
ORe/R/VR	67.1	1.31 (0.72–2.37)	69.4	*1.53 (0.96–2.43)*	48.8	1.03 (0.66–1.59)	38.4	1.14 (0.71–1.84)

	**Self with problem**							

Maj City*	31.9		27.6		13.1		5.2	
IR	35.3	1.17 (0.81–1.69)	34.1	1.35 (0.94–1.96)	15.8	1.25 (0.77–2.01)	8.8	1.78 (0.94–3.37)
ORe/R/VR	34.9	1.15 (0.67–1.98)	49.3	**2.54 (1.60–4.03)**	28.3	**2.62 (1.45–4.72)**	5.9	1.16 (0.39–3.45)

	**Provided services to others with problem**							

Maj City*	22.2		18.8		22.0		21.4	
IR	22.1	0.99 (0.66–1.48)	28.7	**1.74 (1.20–2.52)**	24.9	1.17 (0.81–1.69)	26.2	1.31 (0.88–1.94)
ORe/R/VR	18.0	0.77 (0.45–1.31)	25.2	*1.46 (0.77–2.75)*	25.3	1.20 (0.79–1.81)	21.2	0.99 (0.62–1.58)

	**Family/friends with the problem who received help (as % of respondents with family//friends with the problem)**							

Maj City*	85.8		86.2		90.7		86.1	
IR	89.9	1.48 (0.75–2.90)	82.9	0.78 (0.44–1.38)	88.4	0.78 (0.35–1.76)	87.9	1.17 (0.52–2.63)
ORe/R/VR	76.8	0.55 (0.25–1.20)	73.8	***0.45 (0.22–0.91)***	92.7	1.31 (0.41–4.18)	77.7	0.56 (0.24–1.30)

	**Respondents with the problem who received help (as % of respondents with the problem)**							

Maj City*	68.8		80.4		82.4		78.1	
IR	80.0	1.82 (0.89–3.71)	84.6	1.34 (0.70–2.57)	88.6	1.66 (0.53–5.25)	91.7	-
ORe/R/VR	54.8	0.55 (0.21–1.43)	71.1	0.60 (0.28–1.27)	68.3	0.46 (0.16–1.35)	100.0	-

Maj City = Major City, IR = Inner Regional, ORe/R/VR = Outer Regional/Remote/Very remote; *Reference category; CI = Confidence interval. Note: **Bolded data: **Odds Ratios significant (p < .05). ***Bolded italicised***: Odds Ratios significant (unadjusted only). *Italicised:* Odds Ratios significant (adjusted only).

### Effect of locality on self reported symptoms, experience and exposure to mental illness

There were no significant effects of remoteness on self reported 'depression', 'anxiety', or 'nervousness' during the past month. However, inner regional residents were significantly more likely to report irritability (Table 7(ii)).

Table 7(iii) summarises the percentage of participants from each remoteness category with personal experience or close contact with those with a condition similar to that depicted in the vignette. Outer-remote residents were more likely than those in major cities to indicate that they had experienced a problem similar to depression with suicidal ideation. Outer-remote and inner regional residents tended to more often report having friends and family with depression and suicidal ideation and to report having provided services to others with this type of depression. In addition, outer-remote residents were more likely to report having experienced a condition similar to early schizophrenia and inner regional residents to have a family member or friend with a problem similar to chronic schizophrenia.

Table 7(iii) also includes the percentage of cases in which a family or friend, or the person themselves, had experienced a condition and received help. Outer regional residents were less likely to indicate that family or friends with a problem similar to depression and suicidal ideation received help.

## Discussion

The current study is the first national survey to investigate mental health literacy as a function of remoteness of residence. The most salient finding was that mental health literacy is remarkably similar across remoteness categories. Moreover, those differences that were detected were modest. Nevertheless, there were a number of differences or trends of potential interest including differences in recognition of the mental disorder depicted in the vignettes (in favour of inner regional residents), differences in the endorsement of some mental health practitioners (typically greater endorsement by residents living in major cities) and differences in ratings of helpfulness or harmfulness of some interventions (for example, less endorsement of psychotherapy and greater endorsement of alcohol and (for depression) painkillers among rural residents).

### Identification of mental disorder

The finding that inner regional and outer-remote residents were at least as able as residents from major cities to identify the vignettes does not accord with stereotypes of poorer mental health literacy among rural residents. Indeed, inner regional residents were marginally better able to correctly identify the vignettes depicting depression with suicidal ideation and chronic schizophrenia. It could be argued that the inner regional residents' more accurate identification of depression in the vignette with suicidal ideation is due to their greater exposure to this condition in the context of higher rates of suicide in rural regions and the potentially higher salience and impact of suicide within a small, interconnected rural community. This interpretation is consistent with the higher percentage of inner regional participants who reported having provided services to a person with depression and suicidal ideation. However, if higher exposure were responsible, outer-remote residents should also have shown superior recognition of the suicidal ideation vignettes. They did not. With respect to their superior identification of the chronic schizophrenia vignette, inner regional residents tended, more than major city residents, to report that a family or friend had experienced a disorder similar to that depicted. Again, it might be argued that this exposure and the likelihood that schizophrenia is more salient within a small community contributed to the superior identification by the inner regional residents of chronic schizophrenia. However, such an explanation does not explain the pattern of findings among outer-remote residents. They did not show superior recognition of early schizophrenia although they also lived in small communities and reported greater exposure (self) to early schizophrenia.

### Depression

Although there was no evidence of poorer identification of mental disorders among the rural samples compared to city residents, there was some evidence of poorer depression literacy among these groups. Evidence-based treatments for depression include psychotherapy (cognitive behaviour therapy and interpersonal therapy), antidepressants, ECT and physical activity. Inner regional residents were less likely to correctly rate psychotherapy as helpful. Psychologists typically provide the evidence based psychological therapies. However, consistent with findings reported by Goldney, Taylor and Bain [12] in their comparative study of depression literacy in South Australia, both inner regional and outer-remote residents were significantly less positive about psychologists. Conversely, the more remote groups were more likely than their city counterparts to rate drinking alcohol and painkillers as helpful for depression. Neither is an effective treatment for this condition. The one area in which rural residents showed evidence of superior depression intervention literacy related to antidepressants, which outer-remote residents were less likely to rate as harmful. However, this effect attained significance only after controlling for demographic status. Overall, the findings suggest that depression awareness programs in rural areas should focus on communicating that psychotherapy in the form of cognitive behaviour therapy and interpersonal therapy are effective treatments for depression and that such (effective) therapies are often provided by psychologists. Equally, in any focused depression campaign for rural residents, there would be value in communicating that certain interventions may be toxic or unhelpful, particularly alcohol but also pain killers.

One problem with promoting psychotherapies in rural regions is that access to psychologists and other mental health professionals is typically lower in these regions [20,21]. One potential means for overcoming this disparity in access is to provide therapy for depression over the telephone, or the Internet using experts or automated applications, or a combination of these modalities [22,23]. Recent research indicates that practice based CBT delivered by telephone (e.g., using trained practice nurses) is superior to treatment as usual in general practice [24]. Such methods, or a more centralised method of delivering evidence-based treatments by telephone or Internet, could be adapted for use in rural contexts. The fact that over 60% of inner regional and more than half of outer-remote residents endorsed telephone counselling for depression suggests that the telephone may be an acceptable mode for delivering depression help. In addition, given the relatively recent introduction of internet technology, it is encouraging that approximately half of all rural participants endorsed consulting an online expert for depression and accessing online information. Further promoting the use of the telephone and Internet for the delivery of evidence-based therapy for depression may be of particular utility for rural residents.

Interestingly, rural residents were as aware as those from major cities of Australia's public depression awareness initiative and of media programs relating to depression. Indeed, inner regional residents were somewhat more likely to have heard of the initiative, a finding which is consistent with the results of a more recent survey which found greater awareness of the depression initiative among young Australians in rural regions [25]. Together, these findings provide some evidence to support the continued use of national depression and media initiatives for delivering general as well as more tailored depression literacy and help seeking promotion messages to rural residents.

The finding that the prevalence of self-reported current symptoms of depression over the past month is similar in major cities and more remote groups [25] is consistent with findings from surveys of depressive disorder in previous studies [12,26,27]. Moreover, the findings of greater levels of self-reported suicidal ideation and service provision to those with suicidal ideation among more remote Australians accords with the evidence that suicide completions are greater among rural than city dwellers. Communicating that suicidal ideation can be an important indication of a treatable condition (depression) may be particularly important in rural regions.

### Schizophrenia

The finding that the more remote residents were less likely than city participants to endorse the helpfulness of mental health professionals in the treatment of early or chronic schizophrenia is a cause for concern. Psychiatrists in particular are best placed to formulate and review the pharmacological treatment plan for people with schizophrenia. The relatively lower endorsement of the helpfulness of GPs for early schizophrenia is also of concern given the importance of the GP in a rural context. There was mixed evidence with respect to rural residents' knowledge of the appropriate treatment for schizophrenia. Based on scientific evidence, the treatment of first choice for schizophrenia is antipsychotic medication [28]. Although rural residents were as likely as city residents to rate these treatments as helpful for schizophrenia, outer-remote residents were more likely to believe that antipsychotic medications would be harmful for early schizophrenia. The person depicted in the early schizophrenia vignette is younger than the character in the chronic schizophrenia vignette. It is possible that some outer-remote residents are particularly concerned about potential harmfulness of antipsychotics for a young person. If, based on further research this proves to be the case, there may be value in incorporating within remote area mental health awareness campaigns, the message that antipsychotic medications are important tools for early intervention in young people. Moreover, such campaigns would ideally promote the importance of consulting a GP in cases where a problem is suspected, the importance of involving a psychiatrist in the diagnosis and development of a treatment plan for this condition and the role that a rural GP can play in its ongoing management.

The finding that those in outer-remote regions were more likely to correctly recognise that genetic factors can play a role in the development of schizophrenia is not consistent with the previous Canadian study which found that rural residents were less likely to attribute schizophrenia to a biological cause [13]. However, the two studies are not directly comparable. The Canadian study employed an unprompted open-ended question asking respondents to indicate the cause of 'schizophrenia'. The majority of responses comprising the 'biological' category were 'brain diseases'. By contrast the current study used a structured multiple choice format in which the respondent was asked to indicate the likelihood that the problem experienced by the person in the vignette was inherited or genetic. Further research is required to investigate if the effects of remoteness on biological attribution vary as a function of particular biological attributes.

### Limitations

This study has a number of limitations. As we have noted in our previous papers, the low response rate is of concern and the study lacks data on the characteristics of people refusing to participate. In addition, separate data on the response rates for the inner regional, outer-remote and major city samples could not be constructed from the data available. The previous two comparative studies of mental health literacy and remoteness also failed to report this data. Clearly, differential response rates for the rural compared to the city regions could affect the findings since respondent and non-respondent knowledge of mental health may differ. However, since potential respondents were asked to participate in a 'health' rather than a 'mental health' survey, refusal could not have been directly linked to the mental health content of the survey. Secondly, the current study investigated rurality in terms of AGSC remoteness categories, a system which is based on accessibility of services. This system classifies a small percentage of city residents as belonging to the inner regional category. Moreover, as Judd [29] has pointed out, rural communities are heterogeneous and simple conceptualisations of 'rurality' are inadequate. Whether trichotomised as it was in the current study, or not, the AGSC categories do not take into account all the potentially relevant variables that differentiate between different regions and which might be critical for mental health or mental health literacy. Another limitation is that the sample size of outer-remote participants was relatively small. This may have reduced the power of the statistical tests and mitigated against finding significant effects for this group. Conversely, the study employed a large number of statistical tests. This is likely to have resulted in some spuriously significant results, although an attempt was made to address this by focusing on patterns of findings rather than isolated results. Finally, in controlling for the effects of educational level, it was only possible to classify the data into two broad categories. The results may have been different had finer gradations of educational level been employed. As a consequence of the above limitations the study findings must be interpreted with some caution.

## Conclusion

The current findings provide some indicators of the type of material that might best be incorporated into health promotion programs in regional and remote areas. There is no evidence to support the rollout of campaigns which are premised on the assumption that rural residents are less likely to recognise mental health problems, although the importance of recognition should not be ignored. Rather, such campaigns, at least in Australia, may be more appropriately and effectively focused on a message that emphasises which interventions are effective and the helpfulness of particular professionals such as psychologists and psychiatrists in the delivery of these. At the same time, there is a need to communicate that alcohol and pain killers are not the solution for depression. Such a message must be accompanied by appropriate access to psychological therapy. Given the critical skills shortage in rural areas, there may value in exploring and promoting a range of models for delivering evidence-based interventions distally.

## Competing interests

The authors declare that all authors have served on committees for *beyondblue: the national depression initiative *and have received grants from the organisation, including funds contributed to the survey reported in this paper.

## Authors' contributions

KMG co-designed the survey, wrote the paper and analysed the data. HC and AFJ co-designed the survey and edited the paper. All authors read and approved the final manuscript.

## Appendix. The vignettes

### (a) Depression vignette (John version)

John is 30 years old. He has been feeling unusually sad and miserable for the last few weeks. Even though he is tired all the time, he has trouble sleeping nearly every night. John doesn't feel like eating and has lost weight. He can't keep his mind on his work and puts off making decisions. Even day-to-day tasks seem too much for him. This has come to the attention of his boss, who is concerned about John's lowered productivity.

### (b) Depression vignette with suicidal thoughts (John)

John is 30 years old. He has been feeling unusually sad and miserable for the last few weeks. Even though he is tired all the time, he has trouble sleeping nearly every night. John doesn't feel like eating and has lost weight. He can't keep his mind on his work and puts off making any decisions. Even day-to-day tasks seem too much for him. This has come to the attention of john's boss who is concerned about his lowered productivity. John feels he will never be happy again and believes his family would be better off without him. John has been so desperate, he has been thinking of ways to end his life.

### (c) Early schizophrenia vignette (John)

John is 24 and lives at home with his parents. He has had a few temporary jobs since finishing school but is now unemployed. Over the last six months he has stopped seeing his friends and has begun locking himself in his bedroom and refusing to eat with the family or to have a bath. His parents also hear him walking about his bedroom at night while they are in bed. Even though they know he is alone, they have heard him shouting and arguing as if someone else is there. When they try to encourage him to do more things, he whispers that he won't leave home because he is being spied upon by the neighbour. They realize he is not taking drugs because he never sees anyone or goes anywhere.

### (iv) Chronic schizophrenia vignette (John)

John is 44 years old. He is living in a boarding house in an industrial area. He has not worked for years. He wears the same clothes in all weathers and has left his hair to grow long and untidy. He is always on his own and is often seen sitting in the park talking to himself. At times he stands and moves his hands as if to communicate to someone in nearby trees. He rarely drinks alcohol. He speaks carefully using uncommon and sometimes made-up words. He is polite but avoids talking with other people. At times he accuses shopkeepers of giving information about him to other people. He has asked his landlord to put extra locks on his door and to remove the television set from his room. He says spies are trying to keep him under observation because he has secret information about international computer systems which control people through television transmitters. His landlord complains that he will not let him clean the room which is increasingly dirty and filled with glass objects. John says he is using these "to receive messages from space".

## Pre-publication history

The pre-publication history for this paper can be accessed here:


